# Indigenous chicken production system in different agro-ecology of Indian Himalayan Region: implication on food and economic security

**DOI:** 10.3389/fnut.2023.1244413

**Published:** 2023-09-07

**Authors:** Mahak Singh, R. N. Patton, R. T. Mollier, N. Pongener, Rekha Yadav, Vinay Singh, Rahul Katiyar, G. D. Singh, Sourabh Deori, Sunil Doley, J. K. Chaudhary, Subhash Babu, H. Kalita, V. K. Mishra

**Affiliations:** ^1^Animal Reproduction and Gynaecology, ICAR Research Complex for NEH Region, Nagaland Centre, Medziphema, Nagaland, India; ^2^ICAR Research Complex for NEH Region, Nagaland Centre, Medziphema, Nagaland, India; ^3^Department of Agronomy, School of Agricultural Sciences and Rural Development, Nagaland University, Medziphema, Nagaland, India; ^4^ICAR Research Complex for NEH Region, Umiam, Meghalaya, India; ^5^Bihar Veterinary College, Bihar Animal University, Patna, Bihar, India; ^6^College of Veterinary Sciences and Animal Husbandry, Central Agricultural University, Aizawl, Mizoram, India; ^7^ICAR-Indian Agricultural Research Institute, Pusa Campus, New Delhi, India

**Keywords:** indigenous chicken production system, Indian Himalayan Region, food security, economic security, ecological resilience

## Abstract

The indigenous chicken production system (ICPS) has several use values and ecosystem services. In the last few years, ICPS has been recognized for its possible contribution to household food security, income generation, wildlife protection, and bettering the women’s lives. This study aimed to collect, for the first time, comprehensive information about ICPS in three different agro-ecologies (tropical, sub-tropical, and sub-temperate) of the Indian Himalayan Region (IHR) and its role in food and economic security of traditional communities. In this study region, ICPS is semi-extensive, providing homegrown feed and temporary night shelter. In sub-temperate agro-ecology, females owned non-significant (*p* = 0.170) more indigenous chicken flocks than males. Households in sub-temperate agro-ecologies had significantly (*p* ≤ 0.001) larger flock sizes and tropical livestock units (chicken-TLU). However, the livestock diversity index (LDI) was significantly higher (*p* ≤ 0.001) in tropical and subtropical agro-ecology. The households in the sub-temperate region highly (*p* ≤ 0.001) valued indigenous chicken because of its survivability and adaptability. In absolute numbers significant (*p* ≤ 0.001) higher numbers of adult birds died in past 1 year in sub-temperate agro-ecology. The mortality rate of adult birds in sub-temperate agro-ecology was 9%, and it was 14 and 15% in tropical and sub-tropical agro-ecologies, respectively. In sub-temperate agro-ecology, larger flock size translated into significantly higher (*p* ≤ 0.001) egg production and subsequently a significant (*p* ≤ 0.001) higher egg consumption per household per month. In sub-temperate agro-ecology, households’ dietary diversity score was significantly (*p* ≤ 0.001) higher. Similarly, the average annual income from ICPS was significantly higher (*p* ≤ 0.001) in sub-temperate agro-ecology and accounted for 18% of household income. ICPS’ marketing chain was relatively short in the sub-temperate region. In all agro-ecologies, indigenous chicken and egg demand was significantly higher (*p* ≤ 0.001) in the winter. ICPS litter is used as farmyard manure, enhancing ecological resilience. In all agro-ecologies, the three most frequently cited obstacles to extending the indigenous chicken production system are illnesses, predators, and a lack of chicks availability. ICPS contributes to food and nutritional security, economic stability, and ecological resilience in this hilly and fragile ecosystem. Even though the system is self-sustaining, management and health interventions can increase production and productivity.

## 1. Introduction

Indigenous food systems are highly productive, sustainable, and equitable. These systems preserve rich biodiversity, provide nutritious food, and are climate resilient (1). Indigenous people’s food systems are rooted in traditional knowledge and customary systems, which ensure their existence and well-being despite several challenges (2). Indigenous food systems are essential for food security and food sovereignty since they are founded on millennia of collected knowledge. These food systems are vital for cultural identity, spiritual well-being, and land stewardship. Indigenous Peoples are reviving their agro-ecological food systems because they are more resilient to climate change and provide more nutritious diets than modern food systems.^1^ One such food system is the indigenous chicken production system (ICPS) which has several use values and ecosystem services (3–5).

The indigenous chicken production system (ICPS) is a climate resilient and sustainable food system in low- and middle-income countries (LMICs), especially in fragile ecosystems (5–7). Indigenous chickens account for 80% of the chicken population in Africa (8), and in India, 35% of poultry products come from the indigenous chicken production system (7). Although their productivity is lower than that of intensively raised birds (9), indigenous chickens contribute to food and nutritional security, economic security, and ecological resilience (3, 5, 10). In Indian Himalayan Region (IHR), the indigenous chickens have been part of the indigenous food system of tribal and indigenous people for centuries (7, 11).

The Indian Himalaya Region stretches 2,500 km across 12 states and covers 53.7 Mha. This is ~17% of the country’s total geographical area. It is home to 52 million indigenous and tribal people. The IHR is a source of food, water, and energy for 1.5 billion people. There are five agroecological zones in IHR, ranging from cold arid to warm and humid. The mean annual precipitation ranged between 150 and 4,000 mm. The average annual temperature fluctuates between 8°C and 22°C (12). Environmental, biological, socio-cultural, and economic variations in the Himalayas have led to the evolution of diverse and unique indigenous food production systems involving crop species, and livestock (13). Indigenous people’s food has animal source food (ASF) to meet their dietary requirements. The indigenous chicken production system is an important enterprise for tribal farmers in the IHR (7, 11). However, of late indigenous food systems in the IHR are threatened by deforestation, climate changes, migration, the introduction of an intensive agricultural production system (14), changes in dietary habits, mono-cropping, commodity crops, soil degradation, and market decline.

Despite its enormous contribution to the nutritional and economic security of the Indian Himalayan Region, the indigenous chicken production system is poorly studied as a food system. There is no information available on the characteristics of the indigenous chicken production system in different agroecologies in the Indian Himalayan Region. Knowledge about the characteristics and management practices (including health and bio-security) of indigenous chicken and its role in food and nutritional security are therefore of value to academicians and policy planners to further improves upon the existing food system. Hence, the present study aims (i) to characterize the indigenous chicken production system in three different agro-ecologies (tropical, sub-tropical, and sub-temperate) of the Indian Himalayan Region along with health and biosecurity status, (ii) to identify the indigenous chicken market chain, (iii) to investigate the role of ICPS in food and economic security of households and (iv) to identify the challenges for increasing the system’s productivity on a sustainable basis.

## 2. Materials and methods

### 2.1. Study site

**Figure 1:**
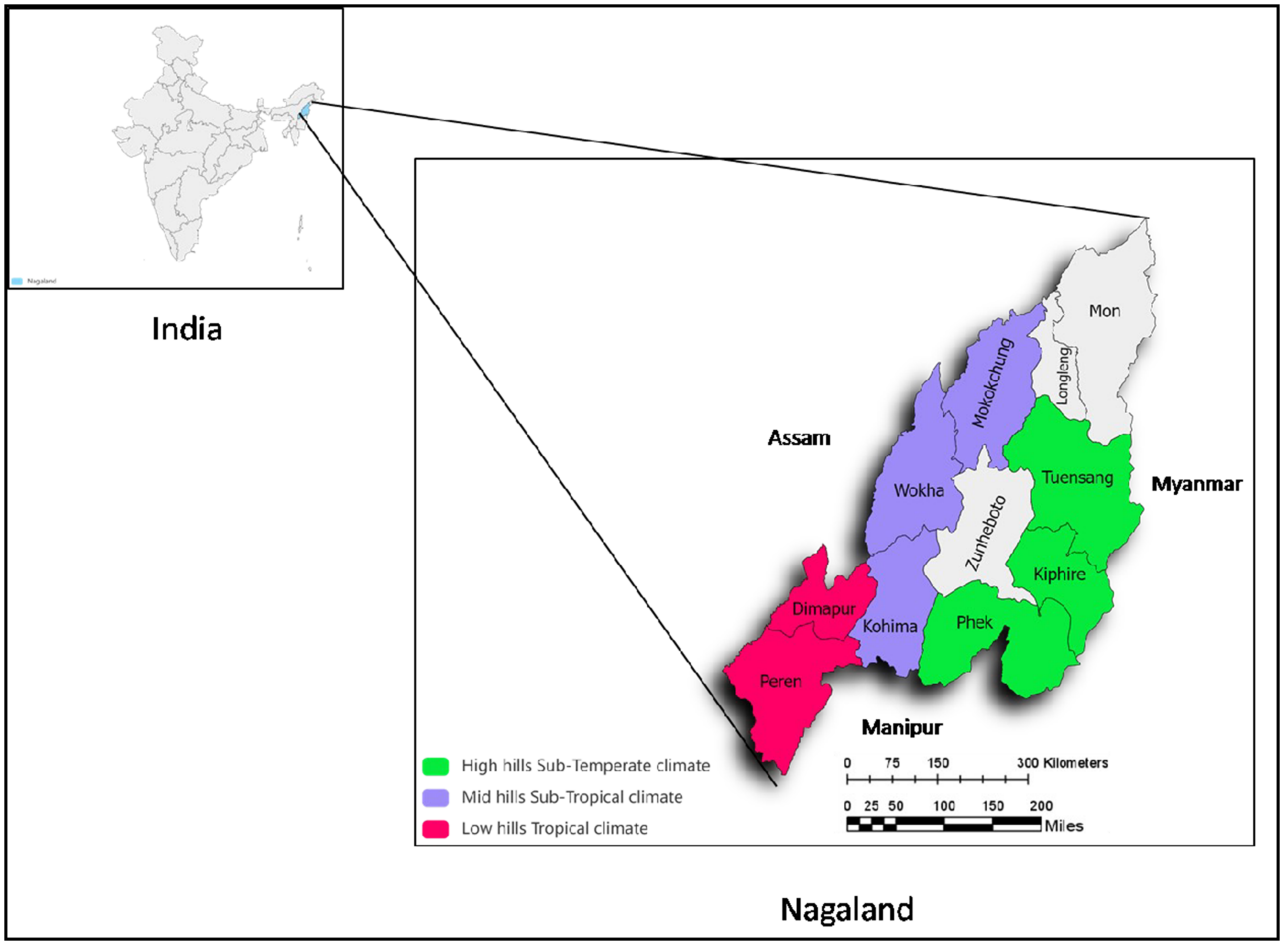
Study location depicting different agro-ecologies in Indian Himalayan Region.

The study was conducted in three agroecologies (tropical, sub-tropical, and sub-temperate) of the Indian Himalayan Region. The study site is located in Nagaland, a Himalayan state in North East India (Figure 1). Table 1 describes three agroecologies, districts, and sample size. Nagaland is a mountainous state of India and lies at 93° 20°E and 95°15°E Longitude, and 25°6° and 27°4°N Latitude with an altitude ranging from 111 to 3,840 m above mean sea level. Nagaland shares an international boundary with Myanmar to its east. Nagaland is inhabited by an indigenous tribal population and has 16 major tribes and numerous minor tribes. In the region, almost 71% of the population depend on agriculture for their livelihood. *Jhum* or slash and burn cultivation is practiced in the study area. Nagaland’s agriculture production system has been close to proto-agriculture, which has enabled close links between nature and people through generations. These traditional practices have been formalized through experiences, and empirical observations, and are deeply rooted in socio-cultural and traditional values.

**Table 1:** Description of different agro-ecologies under study in Indian Himalayan Region (https://statistics.nagaland.gov.in/storage/statistical_data/2021/2811617325911.pdf).

Agro-ecology	Study districts	Households numbers	Description
Low hills tropical climate	Dimapur and Peren	235	Altitude: <500 m above mean sea level,
			Annual rainfall: 1,400 to 1,500 mm mostly during southwest monsoon from May to September.
			Mean monthly temperature: 15°C to 30°C
			Major crops: Rice, Toria, Maize, Soybean, linseed
Mid hill sub-tropical climate	Mokokchung, Kohima and Wokha	192	Altitude: 500 to 1,500 m above mean sea level,
			Annual rainfall:1,400 to 2,000 mm mostly during southwest monsoon from May to September.
			Mean monthly temperature: 11.60°C to 29.10°C
			Major crops: Upland rice, Maize, Coffee, Tea, Tapoica, Cassava, sweet potato
High hills sub temperate climate	Tuensang, Phek and Kiphire	168	Altitude: 1,500 to 2,500 m above mean sea level,
			Annual rainfall: 1,400 to 1,700 mm mostly during southwest monsoon from May to September.
			Mean monthly temperature: 10°C to 19.10°C
			Major crops: Maize, Frenchbean, Upland rice, Soybean, Foxtail millet, Tapoica, Cassava

### 2.2. Sample size and questionnaire development

For sample size calculation, methodology of Thrusfield et al. (15) was used (*n* = Z^2^P(1 − P)/d^2^; where n is the sample size, Z is the statistic corresponding to level of confidence, P is expected prevalence, and d is precision). In each agroecology, the sample size was calculated at 139 based on 70% prevalence (approximately 70% of the population relies on agriculture and related sectors), 99% confidence intervals, and 10% absolute precision. In total, 595 households participated in the study in all three agro-ecologies (Table 1). In sub-tropical and sub-temperate climates, fewer households were studied due to a smaller population, inaccessible terrain, and limited funds. The authors developed a semi-structured questionnaire based on interviews with village chicken owners, field observations, and the relevant literature (10, 16–18). The pilot survey was conducted in two villages using 10 households (five from each village). Following the pilot, the questionnaire was modified to improve clarity. The final questionnaire has eight sections on the socioeconomic status of households, indigenous chicken flock and other livestock on the farm, reason for rearing indigenous chicken, management of indigenous chicken, food and economic security of households, marketing chains of indigenous chicken, health and biosecurity status of the indigenous chicken unit and constraints to expand the indigenous chicken unit.

### 2.3. Study details

Households were selected through a multistage random sampling procedure at the district, block, and village levels. In brief, in each agroecology, eight rural blocks were selected and in each block, two villages were randomly chosen. In the villages, households rearing indigenous chickens were chosen by proportional allocation using a 5% sampling fraction. A pre-tested semi-structured questionnaire was used by the research team to gather the necessary data from the households through face-to-face interviews. Each interview lasted about 40 to 50 min. Data were collected in pen and paper mode, verified by the first author and entered into a Microsoft Excel sheet on the same day. Households verbal consent was requested and granted before data collection began.

The socioeconomic status of households, indigenous chicken management system, marketing chain, and health and biosecurity status of indigenous chicken units were mapped. Data on annual income and income from indigenous chickens were determined after consensus between households and village council members. There may be some biases in income data which cannot be avoided at the field level, however, consensus is the best way to reduce biases (16). A comprehensive inventory of indigenous chicken and other livestock species raised by households was taken at the visit. Chicken Tropical Livestock Unit (TLU) was calculated as per (19). The livestock diversity index was calculated using the Margalef index (20) as it discriminates well and fits well compared to other diversity indices. In addition, it captures a variety of animal species. Household perceptions regarding indigenous chicken rearing were recorded after reaching consensus among adult household members. Most households choose multiple preference indicators.

Data on food and economic security contributed by indigenous chickens were collected on a household recall basis (21). Most of the time, the recall was done by consensus of family members with the adult members of the households being the principal respondents. Egg consumption per month for households and children (less than 6 years of age) was also recorded by the recall. It is well-established that consensus recalls are more accurate and representative than individual responses (16). We also assessed the Household Dietary Diversity score (HDDS) as a measure of food security in the region as per FAO (22) recommendation. The HDDS is meant to reflect, in a snapshot form, the economic ability of a household to access a variety of foods (20). A more HDDS is correlated with caloric and protein adequacy, percentage of proteins from animal sources, and household income. As the indigenous chicken production system is a vital part of the indigenous food system in the region. Therefore, we hypothesized that expanding indigenous chicken flock sizes would increase food and economic security. Households can, however, determine whether their flock is sustainable by learning through experience and observing their constraints. To expand indigenous chicken flocks, we asked households to rank the constraints from most significant (1) to least significant (8).

### 2.4. Statistical analysis

The data were checked and completeness was evaluated. Shapiro–Wilk test was performed to check the normal distribution of continuous data. Descriptive statistics were generated for all the variables in the dataset using SPSS software (Statistical Package for Social Science version 27.0). The results were summarized by questions based on frequency and percentage for categorical variables. The Pearson Chi-square test was used to compare qualitative variables between different agro-ecology. Descriptive statistics analyzed quantitative variables using one-way ANOVA followed by a Tukey’s *post-hoc* test. The difference in mean values for all data analyzed with *p* < 0.05 was considered significant.

## 3. Results

### 3.1. Summary statistics of households’ socio-economic status

Sub-temperate agro-ecologies had a higher proportion (*p* ≥ 0.05) of female respondents (62%) than tropical and sub-tropical agro-ecologies (Table 2). There was no significant difference in respondents’ age between the three agro-ecolgies. The size of families and children were significantly higher (*p* ≤ 0.001) in sub-temperate agro-ecologies. Education levels (*p* ≤ 0.001) and land holdings (*p* ≤ 0.01) were significantly higher in tropical and sub-tropical agro-ecologies. The total annual income of households was similar across the three agro-ecologies (*p* ≥ 0.05).

**Table 2:** Socio-economic description of the households rearing indigenous chicken in different agro-ecologies of Indian Himalayan Region.

Characteristics	Description	Tropical climate (No. = 235)	Sub-tropical climate (No. = 192)	Sub-temperate climate (No. = 192)
Gender of the respondent %	Male	46.8	46.4	38.1
	Female	53.2^b^	53.6^b^	61.9^a^
Age of the respondent	Mean ± SE	46.04 ± 2.57^a^	45.21 ± 3.78^a^	46.91 ± 2.89^a^
Total family size	Mean ± SE	6.04 ± 0.064^b^	5.45 ± 0.060^c^	6.91 ± 0.087^a^
Children	Mean ± SE	3.0 ± 0.05^b^	2.48 ± 0.04^c^	4.07 ± 0.06^a^
Education %	Educated	87.2^a^	76.6^b^	67.9^c^
	Illiterate	12.8^c^	23.4^b^	32.1^a^
Primary occupation %	Agriculture	80	85.4	79.8
	Others	20	14.6	20.2
Land holding (ha)	Mean ± SE	0.75 ± 0.027^a^	0.81 ± 0.044^a^	0.62 ± 0.048^b^
Total annual income (Indian rupees in thousand)	Mean ± SE	42.2 ± 0.07^a^	41.8 ± 0.11^a^	40.7 ± 0.14^a^

Different superscript in a row denotes significant difference at *p* < 0.05.

### 3.2. Households’ chicken flock size and livestock diversification index

**Figure 2:**
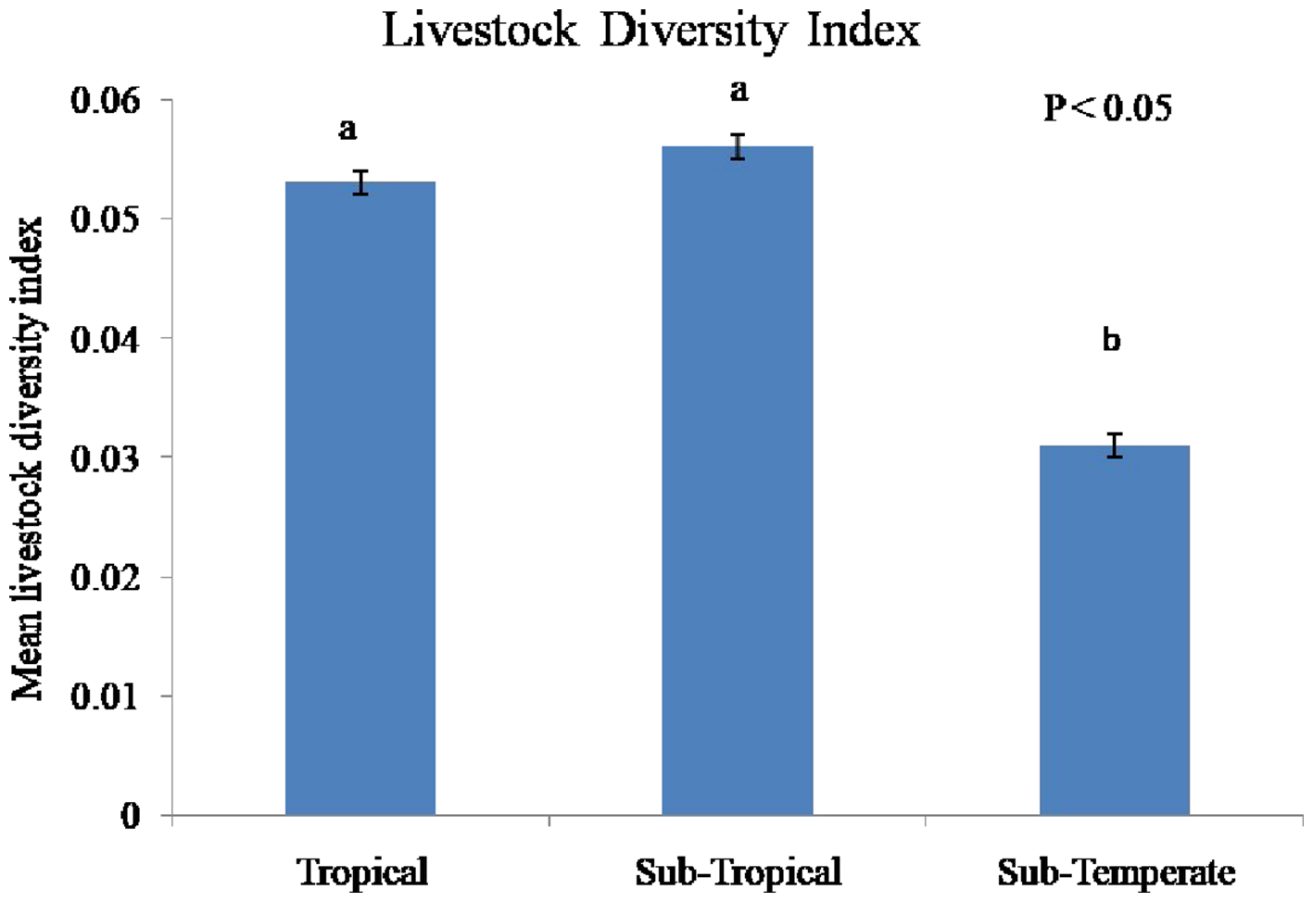
Livestock diversity index in three different agro-ecologies of Indian Himalayan Region. Different letters over bar denotes significant difference (*p* < 0.05).

Analysis of the flock inventory (Table 3) showed that total chickens, as well as individual age group chickens were significantly higher (*p* ≤ 0.001) in sub-temperate agro-ecology. Similarly, the number of chicken tropical livestock units was significantly higher (*p* ≤ 0.001) in sub-temperate agro-ecology. However, livestock types (*p* ≤ 0.001) and livestock diversity index (*p* ≤ 0.001) were significantly higher in tropical and subtropical agro-ecology than sub-temperate agro-ecology (Figure 2).

**Table 3:** Inventory of indigenous chicken reared in different agro-ecologies of Indian Himalayan Region.

	Tropical climate	Sub-tropical climate	Sub-temperate climate
Hen	3.39 ± 0.07^c^	5.41 ± 0.09^b^	8.43 ± 0.19^a^
Cocks	1.84 ± 0.04^b^	1.55 ± 0.04^c^	2.63 ± 0.07^a^
Pullets	2.67 ± 0.06^b^	2.21 ± 0.03^c^	4.35 ± 0.05^a^
Cockerels	2.57 ± 0.05^b^	2.41 ± 0.04^c^	4.49 ± 0.05^a^
Chicks	7.89 ± 0.06^b^	6.98 ± 0.08^c^	11.50 ± 0.18^a^
Total Chicken	18.35 ± 014^b^	18.56 ± 0.11^b^	31.40 ± 0.30^a^
Number of livestock type	2.51 ± 0.04^a^	2.41 ± 0.04^a^	2.09 ± 0.04^b^
Chicken TLU	0.23 ± 0.00^b^	0.24 ± 0.00^b^	0.40 ± 0.00^a^

Different superscript in a row denotes significant difference at *p* < 0.05.

### 3.3. Households’ perception for rearing indigenous chicken

In all agro-ecologies (Table 4), indigenous chickens were valued for their adaptability and survivability, however in sub-temperate agro-ecology (83%), this was significantly higher (*p* ≤ 0.001). Rearing indigenous chicken for meat and egg taste was stated criteria by 31%, 36%, and 41% in tropical, sub-tropical, and sub-temperature agro-ecology, respectively. Scavenging ability and egg production traits were preferred by 44.76% and 42.97% of households in sub-temperate agro-ecology.

**Table 4:** Farmers’ perception (%) for rearing indigenous chicken in three different agro-ecologies of Indian Himalayan Region.

Perceptions	Tropical climate	Sub-tropical climate	Sub-temperate climate
Adaptability and survivability	61.70	65.10	83.33
Meat and egg taste	31.48	36.45	41.66
Less feed and other inputs	19.14	17.70	17.85
Scavenging	29.36	37.29	44.76
Eggs number	22.12	25.20	42.97
Growth rate	12.12	26.25	32.38

### 3.4. Production system of indigenous chicken production system

The majority of households rear indigenous chickens for meat and eggs (Table 5). Most households built their chicken coops using locally sourced materials (bamboo, wood, and grass). The chicken house cleaning was undertaken mostly by females whereas feeding and watering were looked after by both male and female. A significantly (*p* ≤ 0.001) higher proportion of households offered homegrown feed in all the agro-ecologies, however, this was much higher in sub-temperate agro-ecology (93%).

**Table 5:** Management of indigenous chicken in different agro-ecologies of Indian Himalayan Region.

Characteristics		Tropical climate (No. = 235)	Sub-tropical climate (No. = 192)	Sub-temperate climate (168)
Purpose of chicken rearing %	Egg	16.2	14.6	10.1
	Meat and Egg	83.8	85.4	89.9
Type of poultry house %	Temporary	91.9	92.7	92.9
	Permanent	8.1	7.3	7.1
Location of the house %	Backyard	92.3	95.3	92.9
	Front	4.7	2.1	4.8
	Side	3	2.6	2.4
Source of housing material %	Local	92.3	87.5	91.7
	Market	7.7	12.5	8.3
Who looks after cleaning %	Male	13.6	16.7	17.9
	Female	86.4	83.3	82.1
Who looks after feeding and watering %	Male	1.7	1.6	0.6
	Female	9.4	7.3	21.4
	Both	88.9	91.1	78
Type of feed offered %	On-farm	57^c^	68.8^b^	92.9^a^
	Market	11.9^a^	14.6^a^	2.4^b^
	Both	31.1^a^	16.7^b^	4.8^c^

Different superscript in a row denotes significant difference at *p* < 0.05.

### 3.5. Food and economic security contributed by indigenous chicken

**Figure 3:**
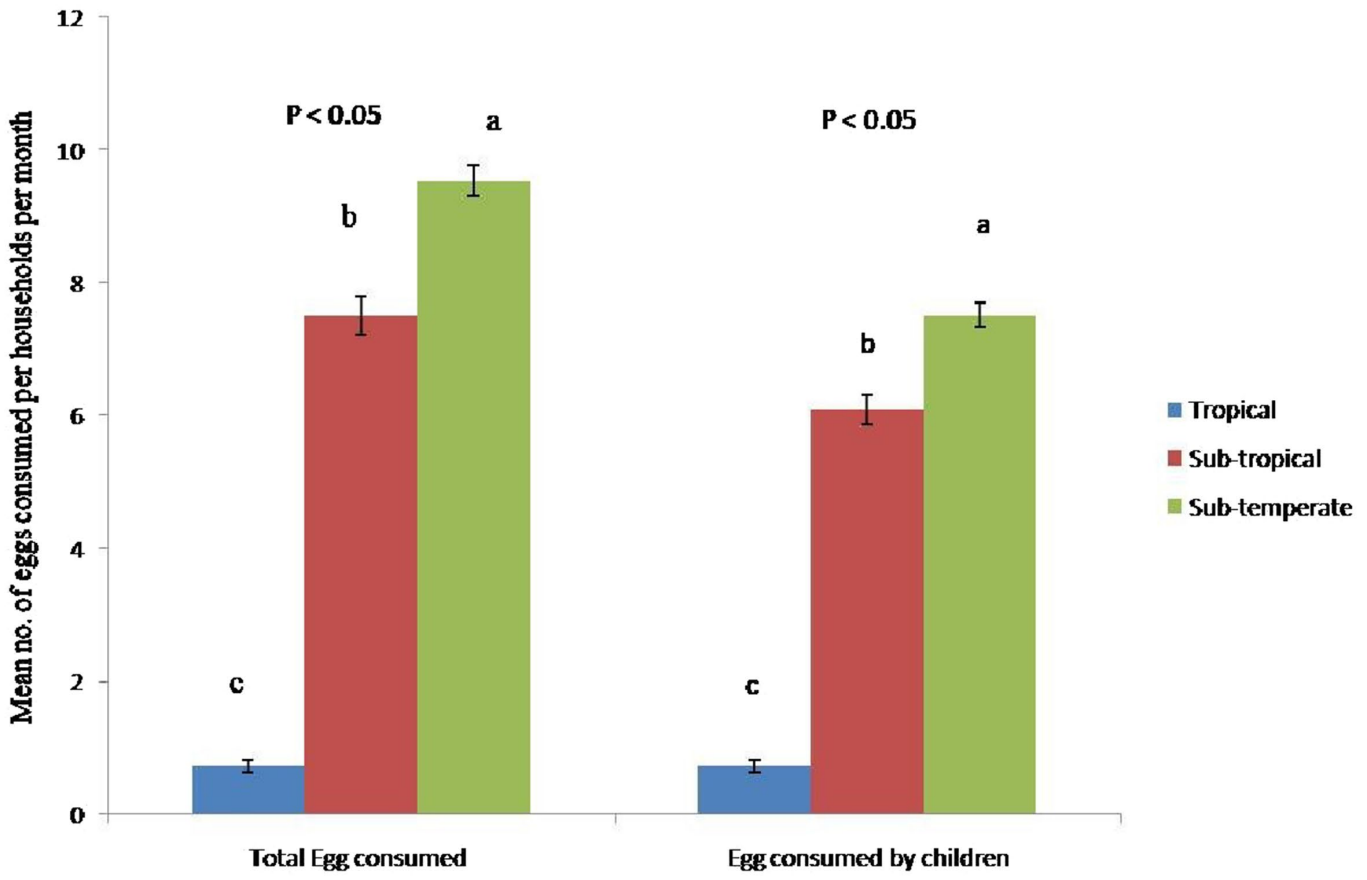
The self-reported number of eggs consumed from indigenous chicken by households and children in different agro-ecologies of Indian Himalayan Region. Letter a,b,c denotes significant difference among groups.

**Figure 4:**
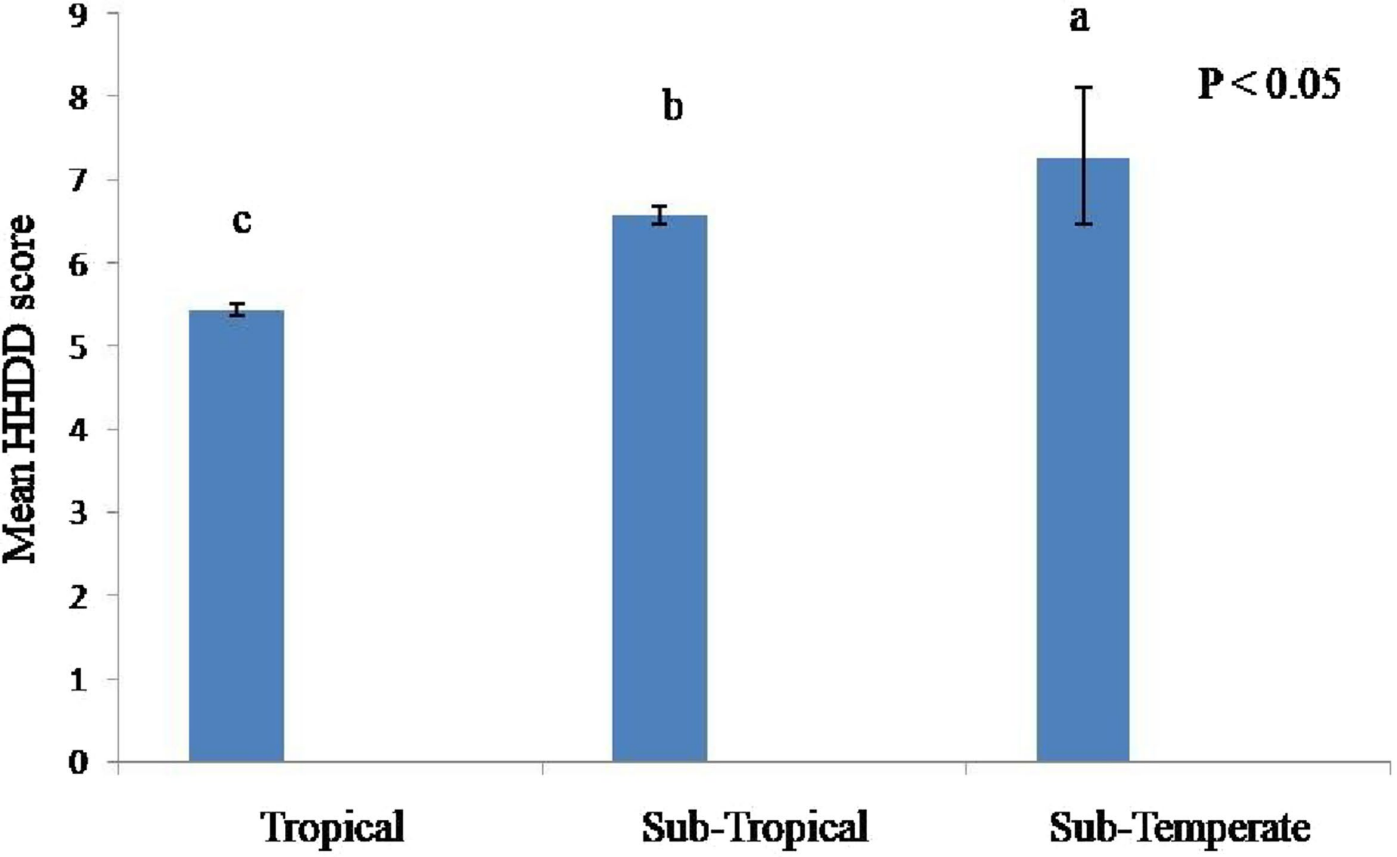
Mean households dietary diversity score in three different agro-ecologies of Indian Himalayan Region. HDDS, households dietary diversity score. Letter a,b,c denotes significant difference among groups.

In sub-temperate agro-ecology, the numbers of eggs produced were significantly higher (*p* ≤ 0.001) than in tropical and sub-tropical agro-ecologies (Table 6). Similarly, households in sub-temperate agro-ecology sold and consumed significantly higher (*p* ≤ 0.001) numbers of indigenous chicken eggs (Figure 3) and adult birds. Significantly higher (*p* ≤ 0.001) numbers of eggs were left for hatching in sub-temperate agro-ecology. In absolute numbers significant (*p* ≤ 0.01) higher numbers of adult birds died in the last year in sub-temperate and sub-tropical agro-ecologies. However, mortality in adult birds constitutes approximately 9, 14, and 15% of the total flocks in sub-temperate, tropical and sub-tropical agro-ecologies, respectively. Survivability of chicks for up to 6 weeks was significantly higher (*p* ≤ 0.001) in tropical and sub-tropical agro-ecologies. Economically, annual income from indigenous chicken was significantly higher (*p* ≤ 0.001) in sub-temperate agro-ecology. Income from indigenous chickens contributed 18, 12 and 10.52% to total households’ income in sub-temperate, tropical and sub-tropical agro-ecology, respectively. The households’ dietary diversity score was significantly higher (*p* ≤ 0.001) in sub-temperate agro-ecology (Figure 4).

**Table 6:** Food and economic security contributed by indigenous chicken in Indian Himalayan Region.

Characteristics	Tropical climate (No. = 235)	Sub-tropical climate (No. = 192)	Sub-temperate climate (No. = 168)
Egg produced in past 1 month	7.88 ± 0.13^c^	15.64 ± 0.36^b^	28.48 ± 0.38^a^
Egg sold in past 1 month	0.17 ± 0.05^c^	1.38 ± 0.18^b^	5.73 ± 0.23^a^
Eggs left for hatching	6.97 ± 0.13^b^	6.72 ± 0.17^b^	13.15 ± 0.20^a^
Hatching percent	85.46 ± 0.37^a^	82.66 ± 0.36^b^	85.45 ± 0.44^a^
Survivability percent (up to 6 weeks)	89.60 ± 0.23^a^	88.44 ± 0.34^ab^	87.51 ± 0.34^b^
Adult birds sold in past 1 year (no.)	9.88 ± 0.49^b^	8.73 ± 0.15^c^	12.24 ± 0.15^a^
Adult birds consumed in past 1 month (no)	3.14 ± 0.05^b^	2.45 ± 0.04^c^	4.55 ± 0.05^a^
Adult bird died in last 1 month	0.33 ± 0.03^b^	0.39 ± 0.04^ab^	0.47 ± 0.04^a^
Birds died in past 1 year	2.59 ± 0.06^b^	2.83 ± 0.07^a^	2.94 ± 0.08^a^
Annual income from Chicken	5.14 ± 0.02^b^	4.4 ± 0.03^c^	7.34 ± 0.04^a^
Contribution of Chicken income to total income (%)	12.18^b^	10.52^b^	18.03^a^

Different superscript in a row denotes significant difference at *p* < 0.05.

### 3.6. Marketing practices of indigenous chicken

The majority of households raised chickens for self-consumption and commercial purposes across the different agro-ecologies (Table 7). Prices of adult birds and eggs were higher in the sub-temperate region. Although the majority of households reported getting a fair price for birds and eggs, the price was significantly higher (*p* ≤ 0.05) in sub-temperate agro-ecology. In the sub-temperate region, most households sold chicken and eggs at local markets, whereas, in tropical and subtropical areas, the home and the local markets were almost equally significant points of sale. In the tropical and subtropical regions, middlemen purchased more chickens and eggs (*p* ≤ 0.001), whereas the majority of households in the sub-temperate region sold chicken and eggs to the consumer. The demand for indigenous chicken and eggs was significantly higher (*p* ≤ 0.001) in the winter season in all agro-ecologies.

**Table 7:** Marketing of indigenous chicken in different agro-ecologies of Indian Himalayan Region.

Characteristics		Tropical climate (*n* = 235)	Sub-tropical climate (*n* = 192)	Sub-temperate climate (*n* = 168)
Purpose of chicken rearing (%)	Self-consumption	11.48	11.45	8.92
	Sale	6.80	2.60	1.78
	Both	81.70	85.93	91.07
Price of adult bird for sale (Indian rupees.)	Cock	500	500	600
	Hen	400	400	450
Price of egg	(Rs.)	10	10	15
Did you get the fair price^d^ (%)	Yes	76.92	82.35	98.03
	No	23.07	17.64	1.96
Sale point (%)	Home	41.34^a^	41.17^a^	16.33^b^
	Local market	58.65^b^	58.82^b^	83.66^a^
Whom did you sold^d^ (%)	Consumer	76.92^b^	73.52^b^	91.50^a^
	Middleman	23.07^a^	26.47^a^	8.49^b^
Highest demand season^d^ (%)	Summer	4.80	2.94	5.22
	Winter	86.53	88.23	84.96
	Rainy	8.65	8.82	19.60

Different superscript in a row denotes significant difference at *p* < 0.05. ^d^*n* = 208 for tropical climate, 170 for sub-tropical climate, and 153 for sub-temperate climate.

### 3.7. Health and bio-security measures of village chicken

Table 8 presents the health and bio-security status of indigenous chicken production systems in different agro-ecologies. The majority of households in tropical and sub-tropical agro-ecologies reported the highest (*p* ≤ 0.001) mortality during the rainy (Monsoon) season. During winter, mortality was significantly (*p* ≤ 0.001) higher in sub-temperate agro-ecology than the other two agro-ecologies. The majority of households did not know about NCD and did not do any vaccination. The majority of households used poultry litter as farmyard manure in all agro-ecologies. Bio-security practices were not followed by most households in all agro-ecologies. The majority of respondents did not follow regular cleaning and disinfection, all-out and all-in systems, isolation of sick birds, and hand washing before and after handling poultry. The majority of respondents reported rodent and wildlife access to feed stores or chicken coops.

**Table 8:** Health and biosecurity measures of indigenous chicken production system in different agro-ecologies of Indian Himalayan Region (%).

Characteristics		Tropical climate (No. = 235)	Sub-tropical climate (No. = 192)	Sub-temperate climate (No. = 168)
Highest mortality in which season (%)	Monsoon	71.1^a^	68.8^a^	50.6^b^
	Winter	26.4^b^	30.2^b^	47.6^a^
	Summer	2.6	1.0	1.8
Do you know NCD (%)	Yes	9.4	9.4	5.4
	No	90.6	90.6	94.6
Did you do the vaccination (%)	Yes	6	7.3	6.5
	No	94	92.7	93.5
Disposal of dead bird (%)	Burial	57	57.3	63.1
	Thrown away	43	42.7	36.9
Disposal of poultry litter (%)	FYM	79.6	81.8	75.6
	No use	20.4	18.2	24.4
Regular contact of birds with children (%)	Yes	81.3	84.9	84.5
	No	18.7	15.1	15.5
Regular cleaning and disinfection (%)	Yes	17	15.6	20.8
	No	83	84.4	79.2
Rodent and wild birds access to Chicken house and feed store (%)	Yes	92.8	88.5	83.9
	No	7.2	11.5	16.1
Hand washing before and after handling chicken (%)	Yes	25.1	14.1	14.3
	No	74.9	85.9	85.7
Isolation of sick bird (%)	Yes	14.5	7.3	5.4
	No	85.5	92.7	94.6

Different superscript in a row denotes significant difference at *p* < 0.05.

### 3.8. Constraints of indigenous chicken production system

Predators, non-availability of chicks, and diseases were the three most reported constraints to expanding the indigenous chicken production system in all agro-ecologies (Table 9). Chicken housing, poor marketing infrastructure, and feed scarcity were the three least ranked constraints by households in the Indian Himalayan Region.

**Table 9:** Constraints to expand indigenous chicken production system in Indian Himalayan Region.

Constraints	Ranking							
	1	2	3	4	5	6	7	8
Predator	188	145	142	85	12	10	8	5
Chicks not available	123	120	115	70	12	12	44	99
Diseases	95	85	80	51	35	80	80	89
Lack of vaccine and medicine	75	70	68	95	92	68	87	40
Inaccessibility to veterinary services	45	40	45	71	111	71	94	118
Feed shortage	43	80	80	87	60	125	81	39
Poor marketing opportunities	20	30	35	80	128	112	97	93
Chicken housing	6	25	30	56	145	117	104	112

Rank 1 = most important, Rank 8 = least important.

## 4. Discussion

The present study characterizes the indigenous chicken production system, along with its contribution to the food and economic security of the tribal communities in the Indian Himalayan region. The percentage of indigenous chicken units owned by women was higher in sub-temperate agro-ecology. It has previously been reported that in less developed regions, indigenous chicken is generally owned and managed by women (7, 17, 23, 24). In sub-temperate agro-ecology, the respondents had less education level. In contrast with the other two agro-ecologies, the sub-temperate agro-ecology is remote, rugged, sparsely populated, and less developed. The primary occupation of the majority of households is agriculture, and their landholding is less than 1 ha. In developing regions, native chicken rearing integrated with agriculture is a century-old traditional livelihood for the rural poor who earn their living on less than 2 ha of land (5, 6, 25, 26).

The total flock size was larger in sub-temperate agro-ecology. Similarly, Chicken-TLU was higher in sub-temperate agro-ecology. However, livestock types and livestock diversity was less in sub-temperate agro-ecology. In contrast to our findings, Haile and Biratu (27) reported that in Ethiopia, farmers in lowland areas keep a higher number of chickens per household than in midland and highland areas. The greater size of indigenous chicken flocks and the lower LDI in sub-temperate agro-ecology indicate a greater reliance on chicken for food and economic security. There is also the possibility that sub-temperate regions might have a higher scavenging feed resource, which could be the reason for the higher chicken population. In all ecologies, chicks make up about 35 to 40% of the flock. The cock to hen ratio ranged from 1.84 to 3.5 in different agro-ecologies. Nonetheless, the flock size per household was less than 50 in all three agro-ecologies. As per the FAO (28) classification, this is an extensive scavenging indigenous poultry production system. In sub-temperate regions, flock sizes tend to be larger, indicating higher genetic polymorphism and, therefore, a wider variety of phenotypic traits and they can serve as the reservoirs of indigenous chicken genetic resources (3). There is high genetic polymorphism in indigenous chickens and this increases with flock size (3, 4), particularly the number of cocks. Households in tropical and sub-tropical agro-ecologies have fewer indigenous birds because they rely on other livestock, as reflected by a higher livestock diversity index. Also, these agro-ecological regions are better connected to major towns in the region and therefore may have easy access to animal-source food. It was earlier reported that the flock size of indigenous chickens varies considerably within and across the regions. This is because of different factors like the purpose of rearing, the socio-economic status of households, etc. (23, 29).

In all three study regions, households prefer to rear indigenous chickens mainly because of their adaptability and survivability, meat and egg taste, and scavenging ability. Indigenous chickens have developed through natural selection in adverse climatic conditions and therefore have accumulated high genetic diversity, making them hardy, resilient to disease, and high survivability (4, 5, 9, 17, 30). The climatic conditions are harsh in sub-temperate agro-ecology and therefore, adaptability and survivability were the main preference criteria for indigenous birds. Native chicken is also preferred because it requires less feed and inputs, produces more eggs, and grows faster. Previous studies have documented that indigenous people prefer native birds because their meat and eggs are tastier, and needed for traditional ceremonial meals (23, 24, 31, 32).

The majority of households rear indigenous chickens for eggs and meat. The management includes a temporary chicken coop for night shelter built with locally available materials. Indigenous birds have multifaceted uses unlike commercial broilers and layers, and they are preferred for egg and meat production (3). Similarly, Kumaresan et al. (11) and Singh et al. (14) reported that indigenous people preferred local birds because of their lean and hard meat. Meat and eggs from indigenous chickens constitute a high-quality food source, densely packed with essential macro-and micronutrients, and play a significant role in the nutritional security of the rural poor (5). Native chickens produce stronger flavored meat with a firmer texture than broiler meat, which makes them highly preferred by traditional communities (29). Households in all agro-ecologies used on-farm production as their primary source of chicken feed, while sub-temperate regions had the highest levels. This is in agreement with earlier reports (5, 11, 33). Traditionally, indigenous chickens live mainly on scavenging and receive minimal supplementary feeding from kitchen waste or household farm produce (5, 11, 23). Besides, scavenging helps indigenous chicken to express their natural behavior, which enhances their welfare.

The present study found that indigenous chickens are a vital part of traditional communities’ food system, particularly in harsh sub-temperate agro-ecology. The larger flock size in sub-temperate agro-ecology translates into higher egg production and higher egg consumption per household, especially by children compared to other agro-ecologies. The HHDS was also higher in sub-temperate agro-ecologies indicating better food security of the households. Similarly, households in sub-temperate agro-ecology sold and consumed higher numbers of adult birds. Economically, the annual income from indigenous chickens was higher in sub-temperate agro-ecology. In Bangladesh, the rearing of indigenous chickens fetches the highest benefit–cost ratio of 1.71 as compared to commercial broilers (1.22) and commercial layers (1.11) (34). Dumas et al. (18) reported that addressing health and management constraints of indigenous chicken resulted in significant increases in households’ income, food security, and physical health of the community. It has been previously noted that the indigenous chicken production system economically empowers rural communities, regularly supplies the family with readily available animal source food, and generates income (17, 35, 36). Snively-Martinez and Quinlan (37) and Dumas et al. (18) reported that as the flock size of the household increases and reliable productivity is achieved, the purpose of keeping native chickens shifts to sources of income. Owing to the short generation interval, indigenous chicken production quickly generates income and enhances the socio-economic status of households (17). Self-reliant indigenous chickens can be sold quickly to buy food and cover school, clothing, and medical expenses (28, 35, 38). Native chickens convert scavenging feed resources and kitchen scraps into high-quality protein and these birds do not directly compete with humans for food (5), which is advantageous from a food security standpoint. Hatching percent and chick survivability up to 6 weeks were better in all three agro-ecologies. Desta (3) reported that indigenous hens are excellent mothers, and in this production system, artificial hatchery and brooding facilities are absent.

In the sub-tropical region, the majority of households sold chicken and eggs in local markets, whereas the home and the local market were almost equal sale points for households in the tropical and sub-tropical regions. In the study region, the weekly market operated in a small town, a big village, or a cluster of three to five small villages. Local residents sell agricultural, vegetable, and chicken produce (live chickens and eggs) in these markets (39). These markets attract buyers from outside the regions, mostly traders and businessmen who purchase these items and resell them in major towns at a premium price. There was a higher proportion of chicken and eggs purchased through middlemen in tropical and subtropical regions. In contrast, the majority of sub-temperate households sell directly to consumers. Tropical and sub-tropical regions are better connected to major towns and have a well-developed transportation system, making them accessible to middlemen and traders. In all agro-ecologies, there was a higher demand for indigenous chicken produce in winter and the majority of respondents reported getting a fair price for poultry and eggs. Although there is no clear-cut seasonality of chicken products in the study regions, the higher demand during winter may be because of the festive season, as the majority population is Christian and November to January is the festive season. The fair price may be because of the high demand for meat and eggs from indigenous chickens, because of their suitability to local taste preferences and perceived health benefits (5, 29, 40). Jin et al. (41) reported that the meat of indigenous chicken in Japan fetches two to five times higher prices compared to broiler meat. Also, the marketing chain was short in all the study regions, and households directly sold to consumers, which maximized their profits.

Approximately 9% to 15% of adult birds died in the last year across the three agro-ecologies. Sambo et al. (29) reported higher mortality in the wet and winter season in native chickens because of infectious diseases. Mortality because of diseases is common in densely inhabited areas where flocks are frequently intermixed, birds are relatively numerous and health prophylaxis and bio-security are least applied. Households have scant knowledge of health and bio-security applicable to chicken farms. The indigenous chickens were in regular contact with family members, especially children, which increased the chance of spreading zoonotic diseases, particularly Salmonella infection (6, 42). Households mostly used poultry litter as farmyard manure. Although high mortality was not reported in the last year in the study area, households reported large-scale mortality in the past among indigenous chickens in the entire village. It was earlier reported that the most common cause of high mortality among village chickens was Newcastle disease (28, 43). During our study, it was also observed that some households reared broilers and improved birds, which might have spread infectious diseases to indigenous chickens. Small-scale chicken growers may choose not to implement bio-security measures because of a lack of awareness of the potential risks or a belief that the benefits do not outweigh the costs (38).

In the present study area, predators, non-availability of chicks, diseases, and non-availability of vaccines were critical production constraints reported by households. Predation is a significant constraint of indigenous chicken production systems, particularly in forest areas, and leads to significant flock loss. Desta (3) argues that as prey animals, indigenous chickens form the local food chain system, and predation is an unavoidable threat. The indigenous chicken production system does not have artificial hatcheries and, for replacement stock, households rely on self-production of chicks. In this system, a hen typically lays eggs then incubates, hatches, and broods the chicks and this cycle takes 105 to 140 days (44) which limits the production of eggs and therefore the number of chicks. Improvement in day-old chick supply to better match farmers’ demand would benefit village chicken production (29). Few households reported diseases as the major constraint. Indigenous chickens are hardy, resilient, and evolved in tandem with the production ecosystem (3, 43). In an earlier study, Scherf (45) reported that the Tanzanian chicken landrace “Mwanza” possesses enhanced tolerance to New Castle Disease. However, further studies are needed to substantiate the disease resistance traits of these ecotypes. Feed, marketing, and poultry housing were the least regarded as constraints in the study area by the households. This indicates the wide range of natural feed resources available in the region.

### 4.1. Conceptual framework regarding multiple use values of indigenous chicken production system

**Figure 5:**
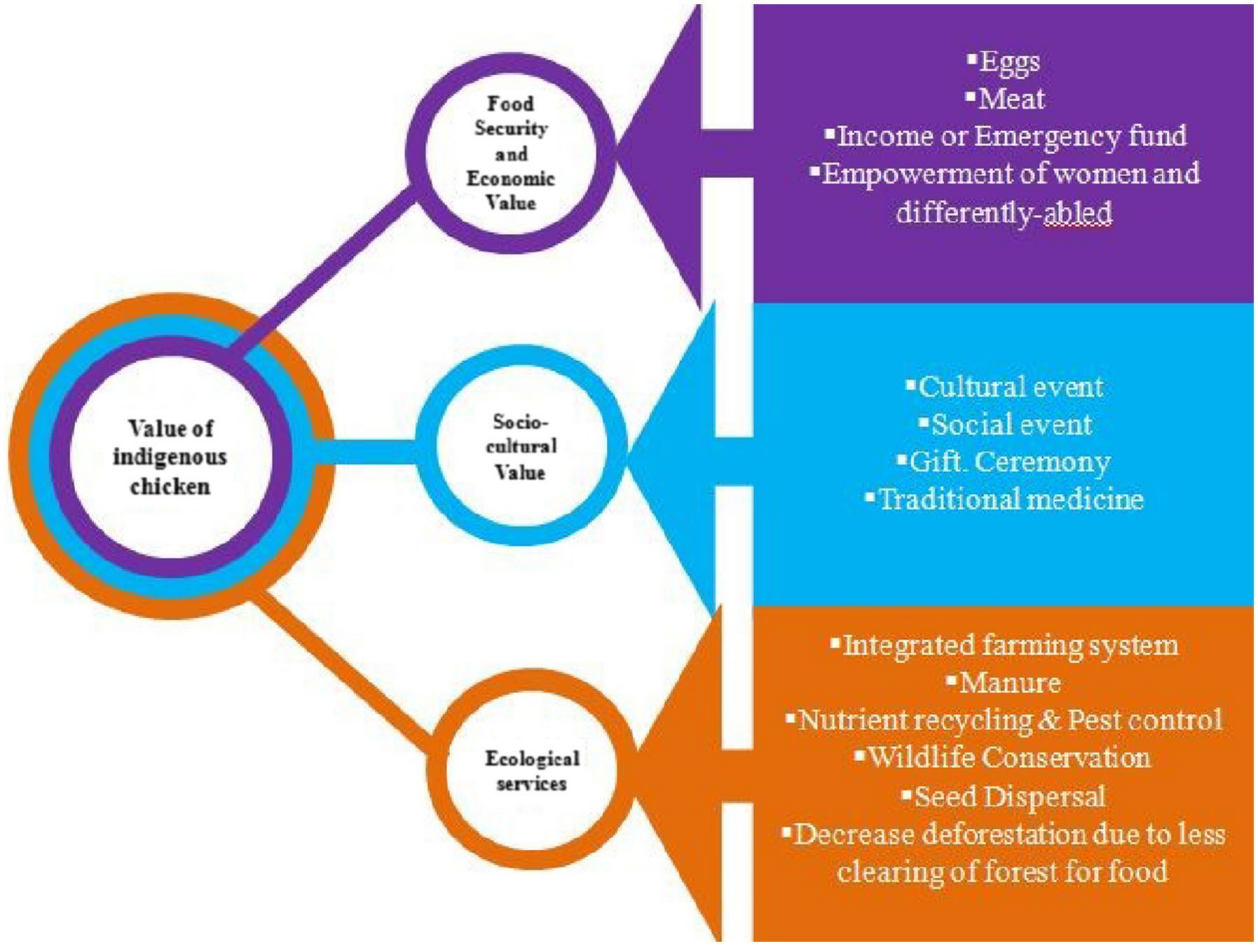
Conceptual framework illustrating multiple use values of ICPS in the Indian Himalayan Region.

A conceptual framework illustrating the multiple-use values of ICPS in the study region is depicted in Figure 5. The study revealed that ICPS has multi-functional use values in the study region. It directly contributes to food and nutritional security by providing eggs and chicken meat to households. Chicken eggs and meat are densely packed with nutrients and are a source of animal food. ICPS income is a significant contributor to the total income of households in sub-temperate agro-ecology. Most of the time, this income is used for children’s education, family health expenditures, or for the purchase of other food items. As most chickens in the study region are managed by women, this contributes to gender equality in male-dominated traditional societies. ICPS’ socio-cultural values include its uses in cultural and social events, gifts, ceremonies, and traditional medicine. ICPS ecological services include genetic diversity, integrated farming systems or mixed farming, manure, nutrient recycling, pest control, wildlife conservation, seed dispersion, and reducing deforestation. The indigenous food system practiced by communities has evolved over a long period in tandem with the ecosystem change and as a result, is resilient and sustainable.

## 5. Conclusions and future directions

The present study characterized the indigenous chicken production system in three different agro-ecologies of the Indian Himalayan region. It also evaluated its contribution to the food and economic security of the tribal populations. Indigenous chickens are an important part of traditional communities’ food systems, particularly in sub-temperate agro-ecology. The ICPS is an extensive scavenging production system. Households in all agro-ecologies use on-farm production as their primary source of chicken feed. In most cases, women are responsible for poultry rearing, which indicates its role in women’s empowerment. The greater size of indigenous chicken flocks and the lower LDI in sub-temperate agro-ecology indicate a greater reliance on chicken for food and economic security. The larger flock size in sub-temperate agro-ecology translates into higher egg production and subsequently higher egg consumption per household, especially by children. The HHDS was also higher in sub-temperate agro-ecology, indicating higher food security of the households. Economically, the annual income from the indigenous chicken was higher in sub-temperate agro-ecology. In all three study regions, households prefer to rear indigenous chicken mainly because of their adaptability and survivability, meat and egg taste, and scavenging ability. The marketing chain was short in all the study regions and households directly sold chickens and eggs to the consumers, which maximized their profits. Households have little knowledge of health and bio-security applicable to the chicken farm. Predators, non-availability of chicks, diseases, and non-availability of vaccines were critical production constraints reported by the households.

The study indicates the significant contribution of ICPS to the food and nutritional and economic security of traditional communities in the fragile agro-ecologies of the Indian Himalayan Region. Considering its multiple-use values and ecosystem services, there is an urgent need to address its critical constraints to further improve its productivity. Also, the climate-resilient traits and genetic diversity of these ecotypes need to be studied through genomics for future use in specific breeding programs. Policy intervention is needed to prevent the genetic erosion of indigenous chicken genetic resources in these areas due to the introduction of exotic chicken germplasm. Depending on the ecosystem’s carrying capacity, some households can be promoted to small-scale semi-intensive ICPS, which will increase incomes and meet growing demand. This will enhance the economy of scale. Furthermore, the health and bio-security aspects of ICPS need to be strengthened to maximize its gains.

## Data availability statement

The original contributions presented in the study are included in the article/supplementary material, further inquiries can be directed to the corresponding author.

## Ethics statement

The animal studies were approved by Institute Animal Ethics Committee, ICAR Research Complex for NEH Region, Umiam, Meghalaya, India. The studies were conducted in accordance with the local legislation and institutional requirements. Written informed consent was obtained from the owners for the participation of their animals in this study.

## Author contributions

MS, NP, RP, and RM: conceptualization. MS, RY, RK, SDe, and VS: methodology. MS, NP, VS, and JC: formal analysis and investigation. MS, SB, and RP: writing—original draft preparation. MS and SDo: writing—review and editing. MS: funding acquisition. HK and VM: supervision. All authors contributed to the article and approved the submitted version.

## Funding

This work was supported by Indian Council of Agricultural Research, New Delhi through ICAR-Poultry Seed Project, ICAR Nagaland Centre (PIMS Code: OXX01915).

## Conflict of interest

The authors declare that the research was conducted in the absence of any commercial or financial relationships that could be construed as a potential conflict of interest.

## Publisher’s note

All claims expressed in this article are solely those of the authors and do not necessarily represent those of their affiliated organizations, or those of the publisher, the editors and the reviewers. Any product that may be evaluated in this article, or claim that may be made by its manufacturer, is not guaranteed or endorsed by the publisher.

## Footnotes

^1^ https://www.iied.org/indigenous-peoples-food-systems-hold-key-feeding-humanity
